# Treatment of Semiconductor Wastewater Containing Tetramethylammonium Hydroxide (TMAH) Using Nanofiltration, Reverse Osmosis, and Membrane Capacitive Deionization

**DOI:** 10.3390/membranes13030336

**Published:** 2023-03-14

**Authors:** Juyoung Lee, Song Lee, Yongjun Choi, Sangho Lee

**Affiliations:** 1 School of Civil and Environmental Engineering, Kookmin University, 77, Jeongneung-ro, Seongbuk-gu, Seoul 02707, Republic of Korea; jyl20101530@gmail.com (J.L.);; 2 Desalination Technologies Research Institute (DTRI), Saline Water Conversion Corporation (SWCC), WQ36+XJP, Al Jubayl 35417, Saudi Arabia

**Keywords:** membrane capacitive deionization (MCDI), semiconductor wastewater, tetramethylammonium hydroxide (TMAH), reverse osmosis (RO), nanofiltration (NF)

## Abstract

As the semiconductor industry has grown tremendously over the last decades, its environmental impact has become a growing concern, including the withdrawal of fresh water and the generation of harmful wastewater. Tetramethylammonium hydroxide (TMAH), one of the toxic compounds inevitably found in semiconductor wastewater, should be removed before the wastewater is discharged. However, there are few affordable technologies available to remove TMAH from semiconductor wastewater. Therefore, the objective of this study was to compare different treatment options, such as Membrane Capacitive Deionization (MCDI), Reverse Osmosis (RO), and Nanofiltration (NF), for the treatment of semiconductor wastewater containing TMAH. A series of bench-scale experimental setups were conducted to investigate the removal efficiencies of TMAH, TDS, and TOC. The results confirmed that the MCDI process showed its great ability as well as RO to remove them, while the NF could not make a sufficient removal under identical recovery conditions. MCDI showed higher removals of monovalent ions, including TMA+, than divalent ions. Moreover, the removal of TMA+ by MCDI was higher under the basic solution than under both neutral and acidic conditions. These results were the first to demonstrate that MCDI has significant potential for treating semiconductor wastewater that contains TMAH.

## 1. Introduction

The exceptional situation of the pandemic has brought about a great change in the trend of all industries as well as our lifestyle for the past two years. Non-face-to-face services that utilize the advancement of technology to provide services or products without direct contact with customers, also known as “no contact”, have prevailed; moreover, the “on-tact”, which means business by online, has emerged. As a result, the market size of information technology is flourishing, and therefore related industries such as display and microelectronics are benefiting. Naturally, the semiconductor manufacturing markets are entering the golden age, and it has been reported that the size of the global semiconductor market was estimated to be USD 43.6 billion in 2021 and is expected to reach USD 115.78 billion or more by 2030, growing at a projected CAGR of 11.5% from 2022 to 2030 [1]. Numerous processes and sub-processes in a manufacturing facility (fab) require ultrapure water, placing a heavy demand on freshwater resources [2]. In addition, a variety of specialty chemicals are used, requiring wastewater treatment. The microelectronics industry generates large amounts of wastewater containing organic contaminants such as acetic acid and tetramethylammonium hydroxide (TMAH), as well as inorganic contaminants such as fluorides and phosphates. [3]. The most frequent chemical used in the etching and photolithography processes of semiconductor production is tetramethylammonium hydroxide (TMAH) [4,5,6].

According to several reports [7,8,9], ensuing biological wastewater treatment procedure could encounter issues due to the presence of TMAH. It is an extremely dangerous substance that is destructive to aquatic life and human health and has acute toxicity. Several fatal poisonings have been reported and the mechanism of action has been linked to neuromuscular toxicity caused by TMAH [10,11,12]. Due to its corrosiveness and toxicity, the presence of TMAH in a natural body of water has adverse effects not only on humans but also on aquatic life and contributes to eutrophication. More information on the acute effects of TMAH is presented in Table 1. However, more than a quarter of a million tons of TMAH solution are reportedly used annually, with the E&S Worldwide industry using more than two million tons annually [13]. In addition, according to a recent estimate, TMAH consumption will increase from USD 950 million in 2018 to USD 1180 million in 2025 [14]. Therefore, the proper and techno-economic treatment of waste water, including TMAH, prior to its disposal is more important than ever.

There exist two primary categories of technologies that can effectively treat effluents containing tetramethyl ammonium hydroxide (TMAH), namely, destruction processes and separation processes. The destruction processes encompass various techniques such as biodegradation (including both aerobic and anaerobic treatment), advanced oxidation processes (AOPs), and hydrodynamic cavitation. On the other hand, the separation processes include adsorption, ionic exchange, electrodialysis, and other membrane-based separation processes. Lei et al. [8] investigated biological remedies for TMAH residual solutions and other sub-stances (monoethanolamine and dimethylsulfoxide). The studies used a combination of aerobic and anaerobic conditions. They confirmed that nitrification can completely convert the ammonium generated during the degradation of MEA and TMAH to nitrate. However, DMSO and TMAH can only be effectively degraded in aerobic and anaerobic environments, respectively. Furthermore, Chen et al. [16] could reject 98% of TMAH by biodegradation in the CANON process, but first anammox had to be grown, and adaptation of the microorganism was difficult. Moreover, the wastewater containing relatively high concentrations of TMAH was reduced by *Mycobacterium* sp. and *Hypomicrobium* sp. [17]. However, at a high initial concentration, nitrification was inhibited.

Chemical oxidation could be used to remove TMAH from wastewater. Hirano et al. [4] developed a method for treating TMAH that combines two novel degradation processes: pyrolysis of TMAH to TMA and selective catalytic oxidation to N_2_, CO_2_, and H_2_O. However, to avoid the creation of hazardous gas, a conversion procedure should be added to convert nitrogen oxides to nitrogen, water, and carbon dioxide. Furthermore, it was showed that the combined UV/H_2_O_2_ and fluidized bed biodegradation methods were able to remove over 95% of TMAH, but the biodegradation of TMAH was difficult with cultures adapted from activated sludge [18].

In the case of the separation processes, Chang et al. [19] proposed the AnOMBR hybrid system to treat low-strength TMAH wastewater. They achieved 99.99% of TMAH rejection by this system, but the phosphorous accumulation in the bioreactor could lead to eutrophication after discharge. Ion-exchange processes have also attracted attention due to their effective performance and relatively inexpensive material [20,21]. Additionally, an improved micellar ultrafiltration technique could be used to separate TMAH from liquid waste [22], and Nishihama et al. [23] established high removal capability under a wide pH range with the zeolite-coated nanofiltration. In addition to pressure-driven processes, thermal membrane processes, i.e., membrane distillation, have also been introduced to remove TMAH [24,25]. However, when the composition of the water is complicated, the efficacy of ion exchange resins rapidly decreases and the filtration processes could suffer due to the short life span of the membranes and the second effluent by the cleaning step.

Since its introduction in the 1970s [26], capacitive deionization (CDI) has attracted increasing interest in both desalination and wastewater treatment due to its sustainable and energy-efficient performance [27]. CDI is a technique that employs carbon electrodes to eliminate dissolved contaminants from aqueous solutions. The CDI process involves the adsorption of cations on the anode surface and anions on the cathode surface by applying a low electric potential difference of 2 V or less. This process occurs in a porous carbon electrode that has a large surface area and excellent electrical current flow, without any electrolysis of water. To regenerate the electrode, the potential difference between the two electrodes is either reduced or reversed, allowing the ions adsorbed on the electrode surface to be discharged back into the aqueous solution. The low electric potential difference required for CDI results in reduced power consumption, and the process is devoid of secondary effluent as it can be regenerated. Nevertheless, the porous carbon electrodes used in CDI can be readily degraded by suspended solids, and the inability to selectively remove them may result in increased energy consumption. To solve these fatal problems, Membrane Capacitive Deionization (MCDI) was introduced in 2006 [28]. The ion exchange membrane (IEM) assists in selective ion adsorption by placing an anion exchange membrane (AEM) in front of the anode and a cation exchange membrane (CEM) in front of the cathode. (M)CDI cells can be combined in several pairs to form a stack to improve ion removal performance. Once enclosed in a housing, these stacks can be connected in parallel or series with respect to fluid flow [29].

Based on the above advantages, MCDI has been used in versatile methods. Choi et al. [30] reported that Ca^2+^ deposition via production of Ca(OH)_2_ on the electrode surface would rapidly lead to a decrease in accessible adsorption sites, resulting in a decrease in CDI performance, and Yoon et al. [31] conducted a study comparing an electrode coated with Ca-alginate to the conventional MCDI and found that it provided reliable outcomes. To selectively recover lithium from mixed multi-cation solutions, Lee et al. [32] reported a modified MCDI system with a carbon electrode for anion capture and a lithium manganese oxide (LMO) electrode for lithium ion capture. Shi et al. [33] reported a successful lithium extraction from brine via MSCDI, which was assembled with a monovalent selective cation exchange membrane. In addition, palladium has been successfully recovered from metal plating wastewater using the MCDI process [34]. However, the focus of previous MCDI research has been on electrodes and IEM development in addition to resource recovery. In the case of semiconductor wastewater, which contains myriad hazardous substances, the attainment of effective removal should be regarded as another significant advantage.

Accordingly, the objective of our study is to investigate the efficient removal of TMAH using MCDI. To achieve this goal, real semiconductor wastewater containing multiple dissolved ions, TOC, and TMAH was obtained from a microelectronics manufacturing company. The wastewater underwent a series of purification tests using MCDI, and the results were compared with conventional processes, i.e., reverse osmosis (RO) and nanofiltration (NF) processes. The removal efficiency of each process was evaluated using a laboratory-scale experimental device. The pH of the solution was also varied to estimate the difference in TOC and TMAH removal in the MCDI process.

## 2. Materials and Methods

### 2.1. Membranes, Electrode, and Chemicals

Thin-film composite membranes made of polyamide (CSM RE4040-BE and CSM NE2540-70) were purchased from Toray Chemical Korea Co., Ltd. for RO and NF processes, respectively. The operating pH range of both membranes was from 3.0 to 10.0 and has the negatively charged surface in this boundary. The IEM was fabricated with pol-yethylene, and its ion penetration capacity was 1.6 meq. /g. The MCDI electrode was composed of graphite and the activated carbon was coated, and it has an adsorption capacity of 16 mg/g (2000 ppm NaCl, 20 mL/min, and 20 °C). Before each experiment, both the IEM and the electrode were soaked with UPW for at least two hours to remove the loaded air inside the pores. Other details of the membranes and electrode are shown in Table 2.

High-purity sodium chloride (NaCl) was purchased from Samchun Chemicals, Republic of Korea. Two types of pH buffers were purchased from Sigma-Aldrich, City, Republic of Korea; the acidic buffer (CAS No. 55965-84-9) had a pH of 2.0 (20 °C in H_2_O) and was a mixture of citric acid, sodium hydroxide, and hydrogen chloride; the alkaline buffer (CAS No. 13840-56-7) had a pH of 10.0 and was composed of boric acid, potassium chloride, and sodium hydroxide. An ultrapure water (UPW) was produced by an ultrapure water system (Human power I+, Human Corporation, City, Republic of Korea) and used for flushing membranes, modules, and experimental devices.

### 2.2. Semiconductor Wastewater

**Figure 1:**
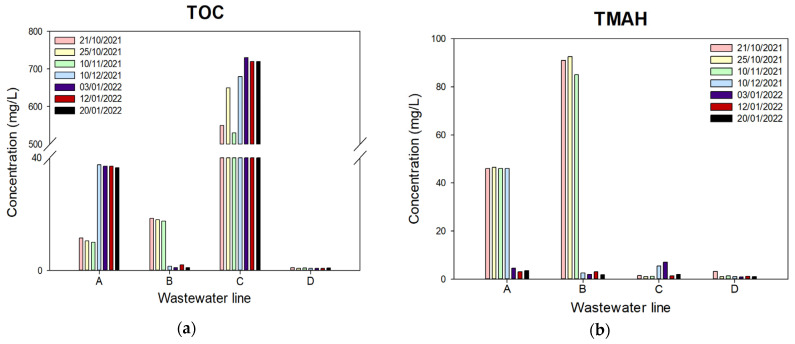
The semiconductor wastewater composition according to collected date and line: (**a**) TOC concentration; (**b**) TMAH concentration.

Real wastewater was obtained from Nepes, which is a semiconductor manufacturing company in City, Republic of Korea; they have four different kinds of wastewater lines A, B, C, and D as shown in Figure 1. According to the water quality analysis report, TOC (Figure 1a) and THAM (Figure 1b) concentrations of these four lines are quite different from each other according to the collection date, as well as ion concentrations, and this company mixes all wastewater before its wastewater treatment process. To minimize the sample variations, we collected the wastewater on a certain day and used it for all the experiments. The compositions of the wastewater are summarized in Table 3.

### 2.3. Lab-Scale Membrane Capacitive Deionization Device

**Figure 2:**
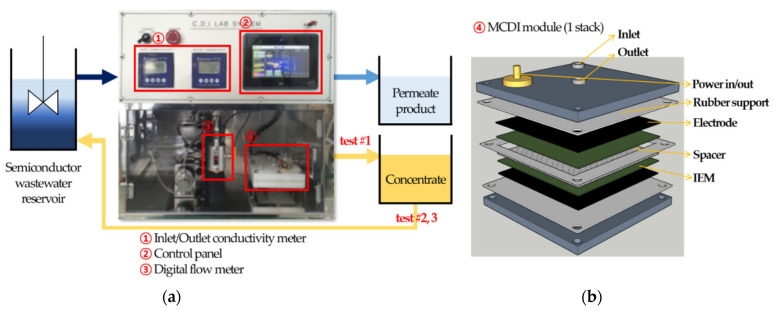

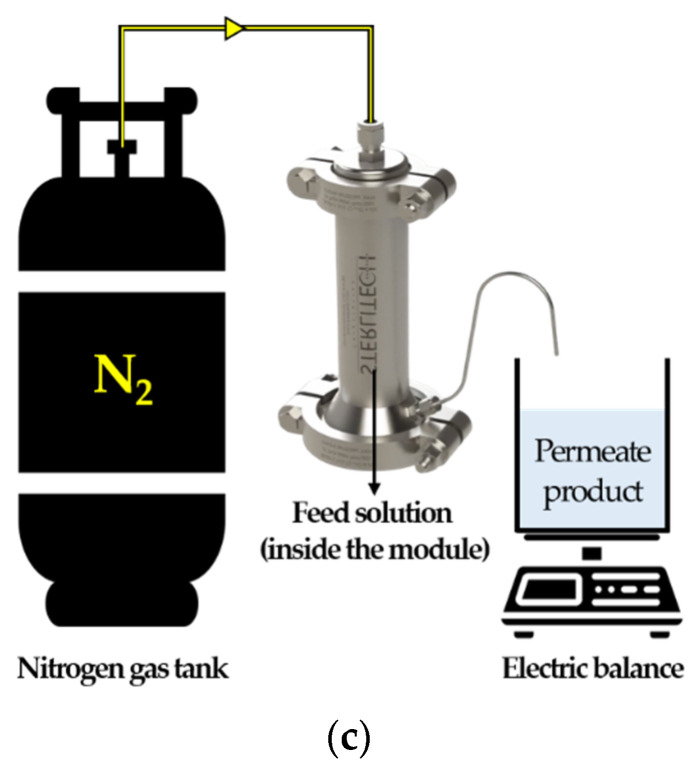
Schematic diagram of lab-scale processes: (**a**) MCDI test procedure scheme; (**b**) detailed design of ion-exchange module; (**c**) RO and NF process scheme.

The laboratory-scale membrane capacitive deionization (MCDI) device was organized and constructed by Pureechem Co., Ltd., Cheongju-si, Republic of Korea; the schematic diagram of the device is shown in Figure 2a. It was operated by a constant voltage (CV) mode with DC power supply, which has 30 V and 25 A capacity. The conductivity sensors were embedded, and their measuring range was from 0.1 μS/cm to 19.99 mS/cm with the cell constant of K = 1. Pressure and flow sensors were also embedded and their measuring ranges were 0 to 5 bar and 5 mL/min to 300 mL/min, respectively. A one-stack fabricated ion exchange module was used as shown in Figure 2b; in this module, three layers of rubber plates supported IEMs, electrodes, and the spacer, and prevented them from excessive compression by the acrylic shell of the module.

### 2.4. Experimental Procedure

RO and NF: Both systems were conducted by stirred cell apparatus (Sterlitech, Auburn, WA, USA). The real wastewater sample was prepared with 20 °C (±1) of temperature adjustment; the initial volume of wastewater was 200 mL, respectively; and the processes were carried out until the recovery reached 90%; permeate product samples were collected and analyzed its conductivity along with TOC and TMAH concentrations.

MCDI: As shown in Figure 2a, three different tests were conducted for the laboratory-scale MCDI process, namely tests #1, #2, and #3. In test #1, both the permeate product generated from the feed step and the concentrate from the discharge step were collected separately; accordingly, its process recovery was only 48%. In test #2, the permeate product was collected as in test #1, but the concentrate was returned to the raw wastewater (feed) and increased the process recovery to 90%. Test #3 was conducted in the same manner as Test #2, but the feed pH was adjusted to 3 and 10 with the buffer to confirm the difference in removal mechanism according to solution pH. Before each test, the control test was conducted with the 500 mg/L NaCl solution to verify the absence of malfunction. The temperature of the semiconductor wastewater was maintained at 20 °C (±1) by the hot plate stirrer (IKA^®^ C-MAG HS7). Solution conductivity and pH were monitored and recorded by the portable conductivity meter set (WTW Multi 3620 IDS, Tetracon^®^ 925 cell for conductivity, and SenTix^®^ 940 cell for pH). All experiments, including MCDI, RO, and NF, were tested at least three times to validate the reproducibility of the results. Additional experimental conditions are summarized in Table 4. After each test, ions, TOC, and TMAH concentrations were analyzed by the TOC analyzer (TOC-L, Shimadzu, City, Japan) and ion chromatography (Dionex ICS-6000 DP, ThermoFisher Scientific Inc., Waltham, MA, USA).

## 3. Results and Discussion

### 3.1. RO and NF Tests: Comparison of Contaminant Removals

**Figure 3:**
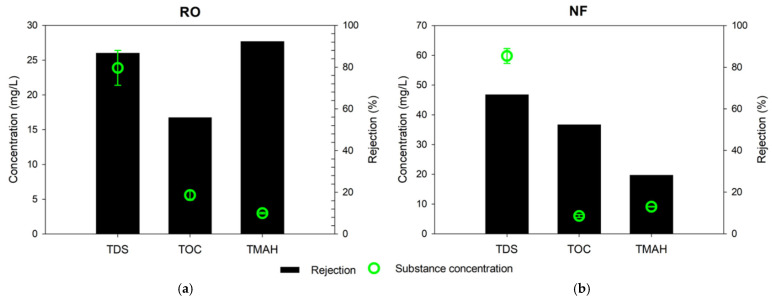
Substance concentration in the permeate product and rejection rate: (**a**) RO; (**b**) NF.

The efficiency of RO and NF membranes in removing contaminants from the semiconductor wastewater was investigated using the stirred cell device. Figure 3 shows the total dissolved solids (TDS), total organic carbon (TOC), and TMAH concentration of the product water from both processes. The TDS, TOC, and TMAH concentrations of the RO product water were 23.9 mg/L, 5.6 mg/L, and 3.0 mg/L, respectively. On the other hand, those of the NF product water were 59.8 mg/L, 6.0 mg/L, and 9.1 mg/L, respectively. The removals of TOC by RO and NF were similar, 56% and 53%, respectively. However, the removals of TDS and TMAH by RO were much higher than those by NF. The removal of TDS by RO was 88%, and THAH was 92%; it was also found that these removals were 20% and 15% higher than those of NF, respectively.

**Figure 4:**
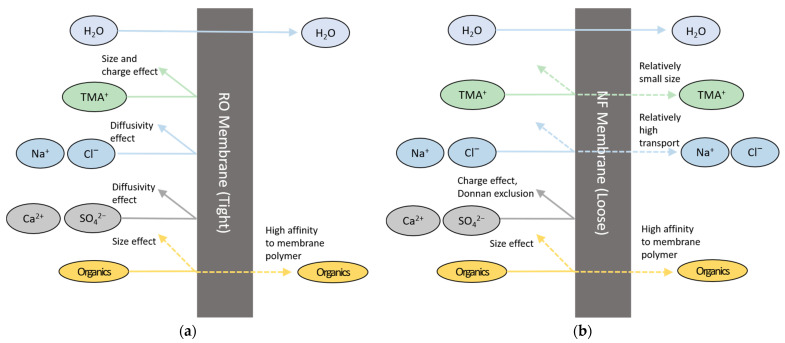
Comparison of rejection mechanisms (**a**) RO; (**b**) NF.

The difference in the removals of TOC and TMAH between RO and NF is attributed to the characteristics of these solutes and the membranes. The organic matters in the wastewater may be either charged or uncharged. In general, RO and NF have higher rejection for charged solutes than uncharged ones [35]. Since TMAH is fully dissociated in water [10,21,36], it has a higher charge than the other organic matters. Therefore, RO can remove more TMAH than the uncharged or slightly charged organic matters in the wastewater. On the other hand, NF cannot have sufficient removal of THAH because its molecular weight cut-off (200–2000 Da) is too large to reject TMAH (molecular weight of 91.15 g/mol). According to previous works, solutes with higher hydration energy show higher rejection by NF [37,38,39]. As shown in Table 5, TMAH possesses low hydration energy, respectively, resulting in its relatively poor rejection of TMAH. Figure 4 summarizes the difference in rejection mechanisms for different solutes by RO and NF. Size and charge effects are important in RO while they are not sufficient to have high rejection of TMAH in NF.

Although RO removed up to 92% of TMAH, the disadvantages of RO technology should be also considered. RO fouling may be a serious issue because the RO feed is the industrial wastewater. Furthermore, RO uses more energy than other conventional processes [13]. The use of NF can reduce the energy consumption, but its TMAH removal is limited as mentioned above. To improve the TMAH rejection by NF, membrane modification may be required [23].

### 3.2. MCDI Test #1: Contaminant Removal with Low Recovery (48%)

**Figure 5:**
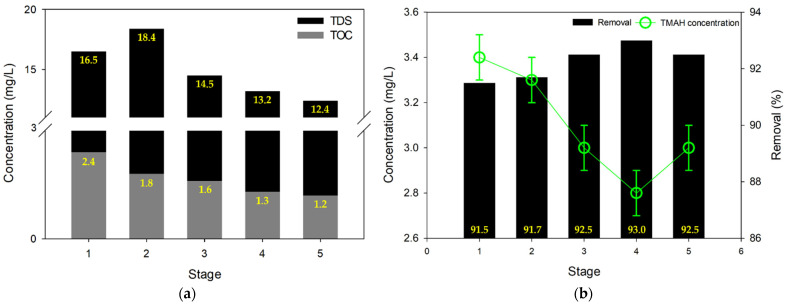
Purification of semiconductor wastewater via MCDI test #1: (**a**) TDS and TOC; (**b**) TMAH concentration.

In addition to RO and NF, MCDI was also considered as a treatment option for the wastewater. The first set of experiments (MCDI Test #1) for MCDI were conducted by separating the product and concentrate (single pass mode), which led to relatively low product recovery (~48%). As depicted in Figure 5a, the TDS of permeate product was decreased gradually as the stage developed. The maximum reduction rate of TDS was 25%, and it implies that the MCDI process requires a certain stabilizing time for better performance. Similarly, the TOC level was also reduced with the development of stages; and it reduced from 2.4 mg/L to 1.2 mg/L. Accordingly, the total concentrations of TDS and TOC of MCDI test #1 were 15 mg/L and 1.7 mg/L, respectively; and it had superior performance compared to formal results of RO and NF. However, in the case of TMAH removal, MCDI showed similar results to the RO process; the final TMAH concentration of MCDI test #1 was 3.1 mg/L, and its removal rate was 92% (Figure 5b). This removal by MCDI is substantially high compared with previous works using different technologies. Chang et al. [51] accomplished 77.6% of TMAH removal via adsorption with activated carbon. Wang et al. [52] reported 89.5% of rejection by degradation with ultrasonic and ozonation processes. Over 99% of successful removal by membrane distillation has also been reported [2]; however, the TMAH content of feed solution was lower than 1 mg/L.

**Figure 6:**
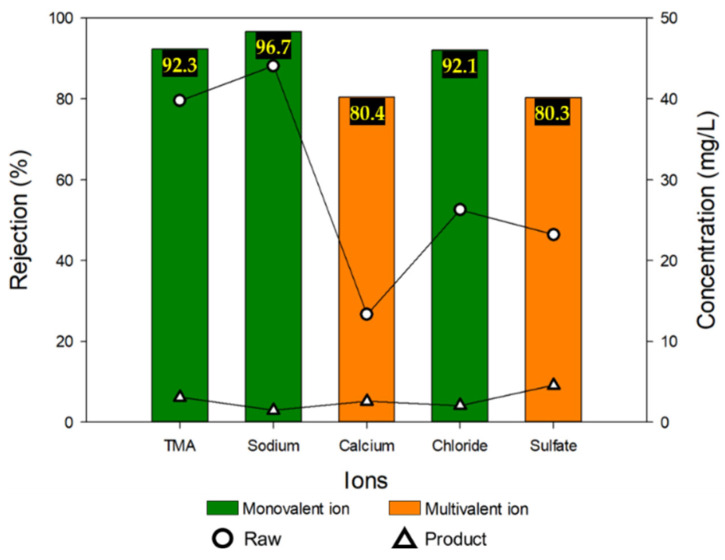
Rejection of both monovalent and multivalent ions including TMA ion in MCDI test #1.

To evaluate the ion electrosorption of the MCDI process, the permeate product was examined by ion chromatography. The initial and permeate product concentrations and the respective removal rates are shown in Figure 6. It is noteworthy that the removals of calcium (Ca^2+^) and sulfate (SO_4_^2−^) ions were lower than those of other ions (TMA^+^, Na^+^, and Cl^−^). The removal of monovalent ions was at least 92%, but that of the divalent ions was only about 80%. These results can be explained by two mechanisms. The first one is the difference in electrosorption rates between the monovalent and divalent ions. According to the literature, monovalent ions generally have higher electrosorption rates than divalent ions due to their weaker EDL overlapping [53,54,55]. This implies that the monovalent ions prefer electrosorption compared to divalent ions, leading to the higher removals. The second one is the difference in the hydration radii. Previous studies report that the electrosorption rates of ions are inversely proportional to their ionic hydrate radii [55,56,57]. As shown in Table 5, the monovalent ions have smaller hydration radii than the divalent ions, which is the reason for their higher removal. Interestingly, TMAH (or TMA^+^ ion) shows lower removal than the monovalent ions (Na^+^ and Cl^−^) but has higher removal than the divalent ions (Ca^2+^ and SO_4_^2−^). This implies that the effect of the atomic valency (monovalent or divalent) is more important than the hydration radius.

### 3.3. MCDI Test #2: Contaminant Removal with High Recovery (90%)

In the previous section, the MCDI process was shown to have considerable ability to remove ions along with TOC and TMAH. However, RO and NF were operated at 90% process recovery, while MCDI recovery was only 48%. Since this is not a fair comparison, another set of MCDI tests were conducted with an increased recovery. In the case of MCDI Test #2, concentrate was returned to the feed stream (recycle mode), and process recovery was improved. As illustrated in Figure 2a, the product was collected in the product water tank and the concentrate was returned to the feed reservoir in this operation mode. As a result, 90% process recovery was achieved in this test.

**Figure 7:**
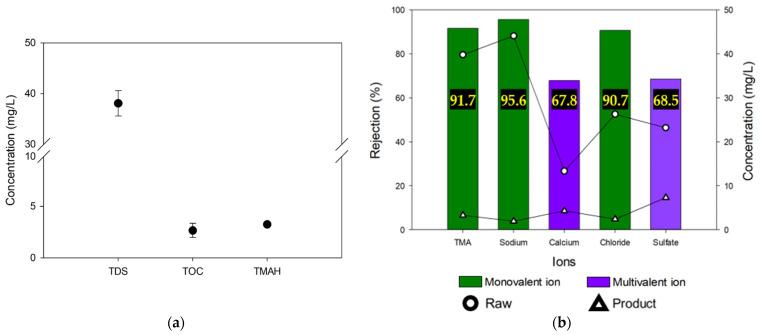
Purification of semiconductor wastewater via MCDI test #2: (**a**) TDS, TOC, and TMAH concentrations of permeate product; (**b**) rejection of both monovalent and multivalent ions including TMA ions.

The water quality of the product water in MCDI Test #2 is shown in Figure 7. The TDS, TOC, and TMAH of the product water were 38.1 mg/L, 2.7 mg/L, and 3.3 mg/L, respectively (Figure 7a). At 90% recovery, the removals of TDS and TMAH were similar to those of RO, but the removal of TOC was higher than the other processes such as RO and NF. The removals of the monovalent ions such as Na^+^, Cl^−^, and TMA^+^ were not significantly affected by increasing recovery. However, the removals of divalent ions such as Ca^2+^ and SO_4_^2−^ were reduced by more than 11% with increasing recovery (Figure 7b). This implies that the removals of the divalent ions are more sensitive to the recovery than the other solutes in the wastewater. Since the electrosorption rates of the divalent ions are lower, their removals appear to be significantly reduced by recycling the concentrate to increase the recovery.

### 3.4. MCDI Test #3: Effect of pH on Contaminant Removal

pH is one of the most important indices in the water treatment process. pH affects the solubility of minerals (hardness removal) and metal salts (coagulation) [53]. Electrostatic interactions between the solutes and surfaces may be influenced by pH [58,59,60]. Here, the effect of pH of semiconductor wastewater on the solute removal efficiency of the MCDI process was investigated. The pH of the semiconductor wastewater was adjusted to 3, 7, and 10 using pH buffer solutions. After pH adjustment, a NaCl solution was added to this wastewater, resulting in an electrical conductivity of 1202 μS/cm (±10). All other procedures were identical to the previous test (MCDI Test #2).

**Figure 8:**
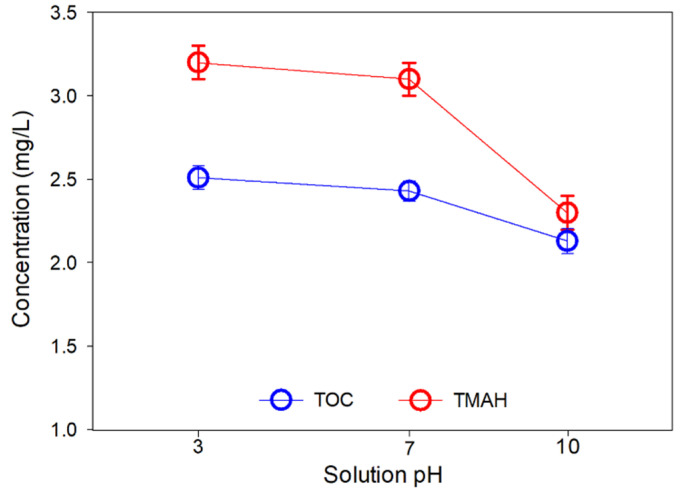
TOC and TMAH concentrations in the permeate product of MCDI test #3 as a function of feed solution pH.

Figure 8 shows the TOC and TMAH in the product water in different pH conditions. The TOC values of pH 3, 7, and 10 feed solutions were 2.5 mg/L, 2.4 mg/L, and 2.1 mg/L, respectively. The removal of three different cases was more than 80%, but the result indicates that the TOC removal efficiency was the highest at pH 10. Interestingly, a similar tendency was observed in the case of TMAH removal. The concentration of TMAH in the product water at pH 3 and 7 solutions was similar to 3.1 mg/L and 3.4 mg/L, but the TMAH concentration at pH 10 was much lower than other cases.

**Figure 9:**
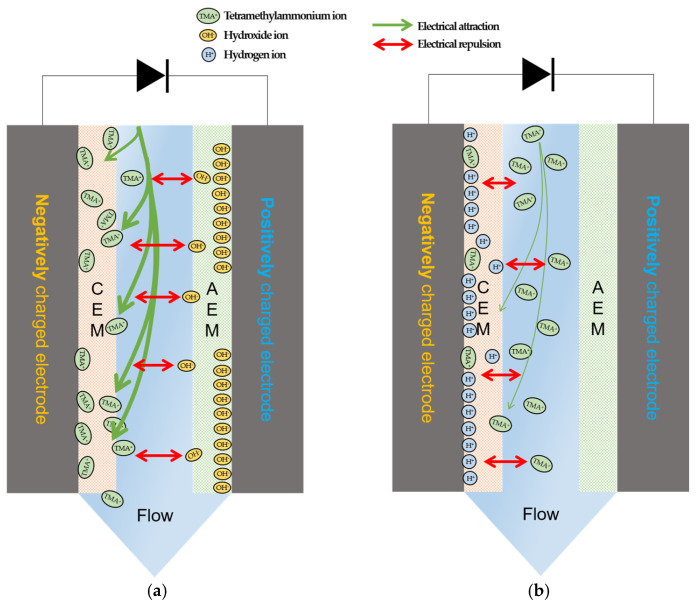
Electrosorption mechanism model of TMA ions in the MCDI process according to the solution pH: (**a**) high pH conditions (pH 10); (**b**) low pH conditions (pH 3).

These results are attributed to the difference in the electrosorption in the electrodes in different pH conditions. As illustrated in Figure 9a, there is a relatively large amount of hydroxide (OH^−^) ions in high pH conditions, which are attracted to the electrode of positive charge. At the same time, TMA^+^ ions migrate to the CEM and the negatively charged electrodes of. Since the zeta potential across the CEM decreases at high pH, TMA^+^ ions can more easily pass through the CEM and be absorbed by the electrodes, resulting in their high removal. On the other hand, the competition of the electrosorption between TMA^+^ and H^+^ ions occurs in low and neutral pH conditions, as described in Figure 9b. Similar phenomena have also been reported in the literature [2,61], confirming the importance of pH adjustment for TMAH removal.

## 4. Conclusions

The present study compares the removal of contaminants in the semiconductor wastewater containing TMAH by RO, NF, and MCDI processes. The major findings of this study can be summarized as follows:

- The NF experimental test shows that it is inadequate to treat TMAH in the wastewater due to its limited molecular weight cut-off;
- The RO process effectively removed TDS ~23.9 mg/L and TMAH ~3.0 mg/L from the semiconductor wastewater. However, this process only accounted for 56% of the TOC removal from the wastewater;
- On the other hand, MCDI showed its sufficient ability to treat the semiconductor wastewater by its adequate removal efficiency of TDS, TOC, and TMAH;
- In addition, it was found that the MCDI process can remove the monovalent ions including TMA ions more effectively than the multivalent ions due to their inherent ionic hydrate radii;
- Furthermore, the TMAH removal capability of MCDI can be improved by pH adjustment. In particular, this improvement was more pronounced when the pH of the semiconductor wastewater was higher.

## Figures and Tables

**Table 1:** Acute effects of the tetramethylammonium hydroxide (TMAH) [15].

Organism	Test Type	Route	Dose
Mouse	LDLo	Subcutaneous	19 mg/kg
Rabbit	LDLo	Intravenous	1 mg/kg
Guinea pig	LD50	Skin	25 mg/kg
Frog	LDLo	parenteral	5 mg/kg

**Table 2:** Membrane and electrode characteristics of RO, NF, and MCDI [15,34].

	RO	NF		MCDI		
				Electrodes	IEMs	Spacer
Material	Polyamide	Poly-piperazine amide	Material	Activated carbon, Graphite	Polyethylene	Polyethylene terephthalate
Water flux	48 L/m^2^-h (at 15 bar)	21.6 L/m^2^-h (at 5 bar)	Capacity	16 mg/g	>1.6 meq/g	-
NaCl rejection	99.7% (at 15 bar)	40–70% (at 5 bar)	Areal resistance	-	<0.5 Ωcm^2^	-
pH range	3–10	3–10	pH range	3–12	3–12	3–12
Roughness (R_rms_)	80.8 nm	1.86 nm	Average thickness	600 μm (±10)	15 μm (±1)	99 μm (±1)
Effective area	7.1 cm^2^	7.1 cm^2^	Effective area	99.2 cm^2^	99.2 cm^2^	-

**Table 3:** Composition of major ions in the real semiconductor wastewater.

	TMAH	TOC	TDS	Na^+^	Ca^2+^	Cl^−^	SO_4_^2−^
Concentration (mg/L)	39.8	12.7	181	44.1	13.4	26.3	23.2

**Table 4:** Experimental conditions of the MCDI test.

Parameter		Condition
Contact duration	Charge	220 s
	Discharge	220 s
	Rest	10 s
Electric potential		±1.3 V
Flow rate		20 mL/min

**Table 5:** Diverse hydration radii and energy of each ion.

Ion	Hydrate Radii	Hydration Energy	References
TMA^+^	~6.3 Å	−160.0 kJ/mol	[40,41,42,43]
Na^+^	2.7 Å	−365.0 kJ/mol	[42,43,44]
Ca^2+^	4.1 Å	−1615.0 kJ/mol	[45,46]
Cl^−^	3.3 Å	−340.0 kJ/mol	[43,47,48]
SO_4_^2−^	3.7 Å	−1016.7 kJ/mol	[46,49,50]
OH^−^	3.0 Å	−510.4 kJ/mol	[46,47,48]

## Data Availability

Not applicable.
